# Dual Nature of Relationship between Mycobacteria and Cancer

**DOI:** 10.3390/ijms22158332

**Published:** 2021-08-03

**Authors:** Marek Fol, Piotr Koziński, Jakub Kulesza, Piotr Białecki, Magdalena Druszczyńska

**Affiliations:** 1 Department of Immunology and Infectious Biology, Institute of Microbiology, Biotechnology and Immunology, Faculty of Biology and Environmental Protection, University of Lodz, Banacha 12/16, 90-237 Lodz, Poland; marek.fol@biol.uni.lodz.pl; 2 Tuberculosis and Lung Diseases Outpatient Clinic, Health Care Facility in Łęczyca, Zachodnia 6, 99-100 Łęczyca, Poland; pkoz@mp.pl; 3 Department of Internal Diseases and Clinical Pharmacology, Medical University of Lodz, Kniaziewicza 1/5, 91-347 Lodz, Poland; jakub.kulesza@icloud.com; 4 Department of General Biophysics, Institute of Biophysics, Faculty of Biology and Environmental Protection, University of Lodz, Pomorska 141/143, 90-236 Lodz, Poland; piotr.bialecki@edu.uni.lodz.pl

**Keywords:** bacilli Calmette–Guérin (BCG), tuberculosis, lung cancer, immune response

## Abstract

Although the therapeutic effect of mycobacteria as antitumor agents has been known for decades, recent epidemiological and experimental studies have revealed that mycobacterium-related chronic inflammation may be a possible mechanism of cancer pathogenesis. *Mycobacterium tuberculosis* and non-tuberculous *Mycobacterium avium* complex infections have been implicated as potentially contributing to the etiology of lung cancer, whereas *Mycobacterium ulcerans* has been correlated with skin carcinogenesis. The risk of tumor development with chronic mycobacterial infections is thought to be a result of many host effector mechanisms acting at different stages of oncogenesis. In this paper, we focus on the nature of the relationship between mycobacteria and cancer, describing the clinical significance of mycobacteria-based cancer therapy as well as epidemiological evidence on the contribution of chronic mycobacterial infections to the increased lung cancer risk.

## 1. Introduction

Lung cancer and tuberculosis (TB) cause millions of deaths worldwide each year. Although 150 years have passed since the identification of the etiological agent of TB—*Mycobacterium tuberculosis* (*M.tb*)—it is not fully understood that chronic inflammation can lead to the development of neoplastic processes. It is believed that this scenario may also apply to pathogenic tubercle bacilli, the presence of which may stimulate the development of lung cancer. On the other hand, the vaccine strain of mycobacteria—*Mycobacterium bovis* BCG (Bacillus Calmette–Guérin)—has been used for decades in the treatment of bladder cancer. These facts indicate the dual nature of the mycobacteria. In this review, the mycobacterial participation in neoplastic (lung cancer) and anticancer mechanisms is described.

## 2. TB and Lung Cancer Epidemiology

Virulent *M.tb*, the causative agent of TB, is responsible for more than 2 million deaths annually, and an estimated one-quarter of the human population worldwide is believed to be asymptomatically infected by the pathogen, a status referred to as latent TB [1]. Active TB (a symptomatic form of infection) develops in about 5%–10% of infected people, mainly in the lungs. Worldwide, TB is one of the top 10 causes of death and the leading cause generated by a single infectious agent (above HIV/AIDS). Globally, an estimated 10.0 million (range 8.9–11.0 million) people fell ill with TB, and a total of 1.4 million people died from TB in 2019 [2]. TB is present in all countries and age groups. Men accounted for 56% of the individuals who developed TB in 2019, women for 32%, and children (aged <15 years) for 12%. The novel virulent coronavirus (CoV) named severe acute respiratory syndrome coronavirus-2 (SARS-CoV-2) causing the coronavirus disease 2019 (COVID-19) pandemic, which is now spreading around the world, may have an impact on the transmission and consequently on TB morbidity and mortality due to greater diagnostic delays, reduced hospitalization, and increased clinical severity. Although the effects of the COVID-19 pandemic are not yet apparent, rigorous monitoring across all national TB centers is needed to prevent the devastating consequences of the infection [3]

**Figure 1:**
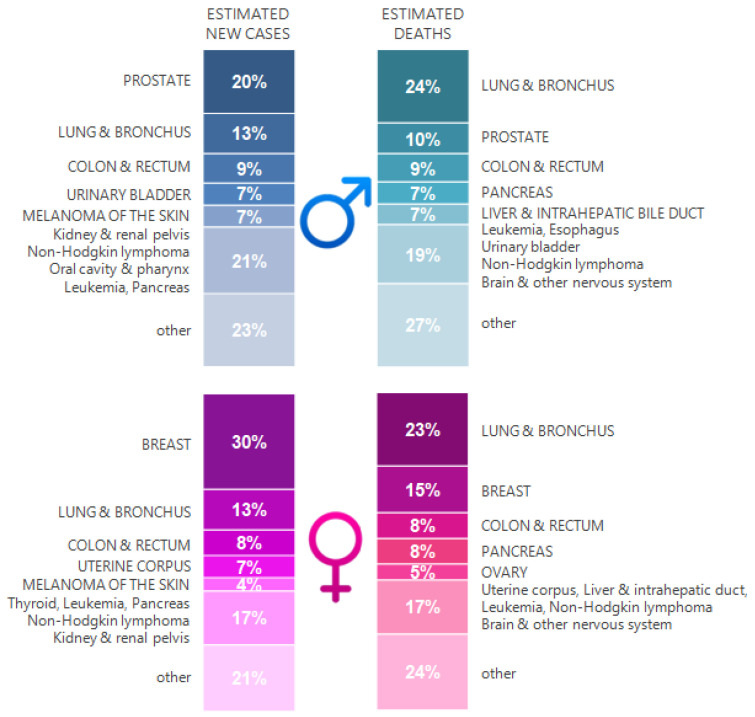
Lung cancer against the background of other cancer types with the highest estimated new cases and deaths by sex, United States, 2020. The estimates are rounded to the nearest 10 and exclude basal cell carcinoma, squamous cell carcinoma of the skin, and carcinoma in situ except from bladder cancer. The ranking is based on modeled forecasts and may differ from the most recent data observed (based on [8]).

Lung cancer is one of the leading malignant neoplasms in the world’s mortality rankings [4]. Global trends show that in the near future, mainly due to the advances in the prevention and treatment of cardiovascular diseases, life expectancy will increase. As a result, cancer is expected to become the main cause of death, as can be seen from the example of epidemiological analyses conducted in many countries, including Poland [5]. Increased exposure to potentially carcinogenic environmental factors may also contribute to the rapidly growing incidence of cancer [6]. Epidemiological data indicate that in 2020, more than 2 million new cases of lung cancer were diagnosed in the world, resulting in 1.8 million deaths, representing 11.4% of the total estimated number of new cancer cases worldwide and 18% of cancer deaths [7]. Lung cancer is the leading cause of cancer morbidity and mortality in men, whereas, in women, it ranks third for incidence after breast and colorectal cancer and second for mortality after breast cancer (Figure 1). It is estimated that in 2020 in the United States (US), 140,730 people died from cancers of the respiratory system—135,720 of them from lung and bronchial cancers (96.4%) [8]. Nearly a quarter out of 606,520 death cases in the US caused by neoplastic diseases per year are therefore a consequence of lung and bronchial cancer [8]. This poses an enormous challenge for the health system and generates a financial burden on society. For comparison, in 2018, the Polish National Cancer Registry received information on almost 167,500 new cases and 101,400 cancer deaths. Malignant neoplasms were the cause of 25.9% of all male deaths and 23.1% of all female deaths [9]. Generally, in Poland, lung cancer is responsible for 30% of all deaths from malignant neoplasms, and the incidence of lung cancer is approximately 20,000 patients annually [10]. Of particular concern is the fact that the percentage of women smoking continues to rise, which is reflected in the percentage of newly diagnosed lung cancer in women [11]. Interestingly, although neoplastic diseases are identified mainly in smoking men, about 15% of lung cancer patients are women who have never smoked [12,13,14].

## 3. Association of *M.tb* Infection with Malignancy Development

Although tobacco smoke is the most influential etiological risk factor in the development of lung cancer [15] (it is recognized that the carcinogenic effects of tobacco smoke can be associated with over 90% of lung cancer cases) [16], the cancerogenic properties are also attributed to radon (colorless and odorless radioactive gas generated by the decay of radium) and asbestos (naturally occurring fibrous silicate mineral commonly used in commercial and industrial settings) [17,18,19]. Another factor that increases the incidence of lung cancer is infection with pathogens causing inflammation, which has been shown to promote carcinogenesis [20]. Human papillomavirus (HPV) infection is detected in 20%−31.3% of lung cancer cases, depending on the tested population. HPV infections are most frequently observed in the Asian population [21,22]. The oncogenic properties of the human immunodeficiency virus (HIV) are also associated with lung cancer [23]. Lung cancer may also be associated with infection by cytomegalovirus (CMV), simian virus 40 (SV40), and polyomaviruses (John Cunningham Virus (JCV), and BK virus (BKV)) [22,24]. The chronic inflammatory process asthma is also considered a factor significantly associated with an increased risk of lung cancer (odds ratio 1.44, 95% CI 1.31–1.59; *p* <0.00001) [25]. It has been documented that pulmonary TB caused by the intracellular pathogen *M.tb* increases the risk and mortality of lung cancer [26,27,28,29,30]. In a cohort study adjusted for comorbidities by Yu et al., the adjusted hazard ratio (aHR) for lung cancer in TB patients was 3.32 (95% CI: 2.704.09) [27]. It was estimated that lung cancer was 11 times more common in TB patients than in non-TB patients, and the association between lung cancer and prior TB (RR 3.43; 95% CI: 1.936.11) was confirmed in a systematic review by Liang et al. [31]. The most recent cohort study involving 20,252 participants in South Korea showed that when compared to the control group, the hazard ratio of lung cancer among individuals with old pulmonary TB was 3.24 (95% CI, 1.875.62). Furthermore, in patients with old pulmonary TB, the hazard ratios of lung cancer for never-smokers, ex-smokers, and current smokers were 3.52 (95% CI, 1.1710.63), 2.16 (95% CI, 0.895.24), and 3.71 (95% CI, 1.499.22), respectively, compared to the control group. This suggests that individuals with old pulmonary TB are at increased risk of developing lung cancer when compared to the general population without pulmonary TB [32]. On the other hand, cancer patients are known to have a higher incidence of TB. The aHR for TB was 1.67 (95% CI:1.421.96) in a retrospective cohort analysis of cancer patients [33]. Esophageal, oral, and lung cancers (6.4, 3.5, and 2.8, respectively) and hematological malignancies had the greatest incidence rate ratio (IRR) of TB (IRR 3.0). In a prospective study, the risk of TB in patients with solid-organ malignancies was found to be almost 4.5 times higher than in the non-malignant group [34].

The association between TB and lung cancer is not fully understood. Lung cancer can develop independently and decrease local immunity, leading to latent TB reactivation or new exogenous infection. Prolonged inflammatory response in TB with substantial remodeling of lung tissue may function as a cause of cancer. Chronic *M.tb* infection has also been linked to cell dysplasia and squamous cell lung cancer (SCC). By generating DNA damage, Mycobacterium-infected macrophages may play a role in TB-induced carcinogenesis. These findings support a causal relationship between TB and malignant transformation [35]. The main rationale for considering a strong association between TB and lung cancer is that carcinoma can develop from TB scars (scar carcinoma), occur by epithelium metaplasia of tuberculous cavities, develop in old TB lesions, and reactivate the old focus of TB [36]. TB-related chronic inflammation and fibrosis can cause genetic mutations and changes. Lung parenchyma tissue is involved in both TB and lung cancer. Furthermore, continuous cough in lung cancer, morphological vascular variations, lymphocytosis processes, and the production of certain immune mediators such as interleukins are factors that have led to the hypothesis that TB plays a role in lung cancer [26,31,37,38,39]. According to some studies, inducing necrosis and apoptosis or reactivating TB, especially in immunocompromised patients, can lead to an increase in interleukin (IL)-17 and tumor necrosis factor (TNF)-α, which can either decrease tumor protein 53 (TP53) activity or increase B-cell lymphoma 2 (Bcl-2) expression, decrease (Bcl-2-associated X protein) Bax expression, and inhibit caspase-3 expression by reducing mitochondrial cytochrome oxidase expression [40,41]. It has also been suggested that after receiving the BCG vaccine, the host immune system is strengthened, with increased levels of interferon (IFN)–gamma (γ), nitric oxide, and IL-2. As a result, CD4^+^ lymphocyte function is boosted, and the anti-cancer immunity is enhanced [42].

In the context of risk factors associated with lung cancer, individual genetic predispositions, including those located at the loci (10q25.2, 6q22.2, and 6p21.32), are not without significance [20]. Molecular changes in lung cancer can be divided into those concerning growth factor receptors (epidermal growth factor receptor (EGFR), fibroblast growth factor receptor (FGFR), mesenchymal epithelial transition factor (MET)), translocations (anaplastic lymphoma kinase (ALK), ROS proto-oncogene 1 (ROS-1), RET proto-oncogene (RET)), activation of oncogenes (Kirsten rat sarcoma viral oncogene homolog (KRAS), v-raf murine sarcoma viral oncogene homolog B1 (BRAF), discoidin domain receptor-2 (DDR2), phosphoinositide-3-kinase catalytic alpha polypeptide (PIK3CA)), inactivation of suppressor genes (tumor protein 53 (TP53), liver kinase B1 (LKB-1), phosphatase and tensin homolog (PTEN)), and epigenetic changes [43].

Another factor that might be considered to play a critical role in the malignancy development in the context of *M.tb* infection can be immunodeficiency defined as an inherited or acquired disorder with defects in the function of the immune system [44]. Genetic disorders underlying immunodeficiency may predispose to oncogenesis due to impaired immune surveillance of the tumor and abnormalities related to the course of infection and/or inflammation [45]. A number of disorders that impair the function of macrophages, dendritic cells, NK cells, and T lymphocytes have been found [46]. Specific mutations in the genes including signal transducer and activator of transcription 1 (STAT1), NF-κB essential modulator (NEMO), interferon regulatory factor 8 (IRF8), GATA binding protein 2 (GATA2), and cytochrome B-245 beta chain (CYBB) have been shown to predispose individuals to mycobacterial infections, including TB [46]. Studies of Mendelian susceptibility to mycobacterial disease (MSMD), a predisposition to clinical disease caused by weakly virulent mycobacteria, such as BCG and non-tuberculous mycobacteria (NTM), confirm the genetic basis of the increased susceptibility to TB [47]. Acquired immunodeficiencies along with immunosuppressive drugs used during the treatment may also predispose to the development of mycobacterial diseases. Childhood leukemia has been documented to be associated with a higher risk of developing TB and BCG or NTM infections following bone marrow transplantation or during chemotherapy [48,49,50]. Moreover, patients treated with immunosuppressive drugs for transplantation or other conditions such as severe aplastic anemia or hemophagocytic lymphohistiocytosis have also been found to have NTM, *M.tb*, and BCG infections [51,52,53,54,55]. However, infection with human immunodeficiency virus (HIV) is the most common risk factor for the development of active TB, leading to high mortality among patients with acquired immunodeficiency syndrome (AIDS) [56]. HIV infection strongly influences the pathogenesis of TB by causing a progressive decline in CD4 T cell immunity, resulting in a higher risk of clinical TB, with more frequent extrapulmonary involvement, and atypical radiographic signs [57]. HIV-positive patients, due to defective T-cell-mediated immunity, may be prone to the consequences of non-tuberculous mycobacterial infections, which can manifest as cavitary or noncavitary lung disease as well as disseminated disease [58].

## 4. Mycobacteria as Causative Agents of Cancer

A growing number of studies indicate an increased risk of cancer associated with chronic mycobacterial infections [27,59,60,61,62]. The connection between pathogen-induced persistent inflammation and tumorigenesis has been documented mainly for M.tb, but also some non-tuberculous species including Mycobacterium avium complex or Mycobacterium ulcerans [63,64,65].

The success of M.tb as a persistent pathogen is largely attributed to its ability to survive in the host tissues. It has acquired various strategies to reside and multiply within macrophages, frontline host immune defense cells, avoiding an acquired immune response or subverting its consequences [66,67]. By suppressing macrophage maturation and lysosomal acidification as well as inhibiting oxidative stress, apoptosis, and autophagy, M.tb is capable of remaining in the host for a long time. The hallmark of chronic tuberculous inflammation is the formation of granulomas comprising aggregates of immune cells, including macrophages, giant cells, and foamy macrophages in the center, surrounded by the lymphocyte-rich marginal zone [68]. The efficient maintenance of granulomas during M.tb infection, requiring the activity of a wide variety of immunocompetent cells, may generate a microenvironment predisposing to malignant transformation [69,70,71]. Many hypotheses based on in vitro and in vivo experiments try to explain the contribution of M.tb-induced inflammatory events to lung cancer development [59,60]. It is generally accepted that the process of tumorigenesis includes several steps: an initiation stage, involving DNA damage; the promotion stage, involving cell proliferation and the fixation of mutations from premutational lesions; and the tumor progression step, involving expansion of the mutant cells and subsequent tumor growth [72,73]. M.tb-associated cancer may arise as a consequence of chronic inflammatory changes that lead to metaplasia of epithelium in the lung caverns, in calcified lymph nodes, and old scars in the bronchi [36]. The most common forms of cancers are cavern carcinoma, carcinoma of the drainage bronchus, and peripheral lung scar cancer [74,75]. The initiation of the tumor development process is a result of attracting macrophages and other cells to the sites of M.tb infection [60,76,77]. A wide range of toxic agents, such as reactive oxygen intermediates, tissue-destructive proteases, as well as prostaglandins, leukotrienes, and cytokines, produced by activated macrophages and other leukocytes, elicit a profound inflammatory reaction leading to tissue damage and genomic alterations [60,76,77,78,79,80,81]. Activation of the proinflammatory pathway mediated by nuclear factor NF-κβ in macrophages and epithelial cells is accompanied by an increase in the proliferation rate of cells with damaged DNA, which in combination with increased angiogenesis stimulated by cyclooxygenase-2 products leads to the initiation of lung tumorigenesis [72,82]. Moreover, repairing the tissue damaged by M.tb-induced inflammatory reactions can lead to the fibrosis and scarring of the lung tissue, which is also linked to an increased risk of lung cancer. Lung tissues infected for years with M.tb undergo multiple processes of inflammation and tissue repair, which generates a favorable environment for tumorigenesis and increases the risk of lung cancer development [59,60,82]. Moreover, M.tb can induce the release of inflammatory mediators, e.g., tumor necrosis factor (TNF)-α and interleukin (IL)-1, IL-2, and IL-12, which can be viewed as cancer promotors [81].

The simultaneous or sequential occurrence of TB and a variety of histological types of lung cancer have been documented in numerous studies; however, the causal link between the two is still not clear [27,31,61,83,84,85,86,87,88]. Nationwide population-based cohort studies performed in Taiwan, the United States, and Lithuania have revealed that pulmonary TB is an independent risk factor for lung cancer [27,61,86,89]. A study conducted in India showed that M.tb lesions were found in the lungs in 30%-33% of patients with tumors compared with 7% in the general population [90]. The development of lung cancer at the site of scars and old TB lesions was also confirmed by other research groups [38,61]. In a mouse model, Nalbandian et al. showed that chronic M.tb infection in the lungs is sufficient to cause a multi-step transformation of cells associated with TB lung lesions through squamous cell dysplasia to malignant squamous cell carcinoma [91]. The authors observed that in addition to DNA-damaging reactive oxygen and nitrogen intermediates, M.tb-infected macrophages within TB lung lesions produced epiregulin, a protein belonging to the epidermal growth factor family, which may participate in the initiation and promotion of lung tumorigenesis. A study by Cao et al. suggested that M.tb might repress Th1 immune response and promote lung cancer metastasis through the programmed cell death protein 1 (PD-1)/programmed death ligand 1 (PD-L1) signaling pathway, while in the opinion of Holla et al. the inhibition of TNF-α-mediated apoptosis via downregulation of the expression of tumor suppressor p53 could be a possible mechanism of mycobacteria-assisted tumorigenicity in type II epithelial cells [92,93].

Mycobacterium leprae (M. leprae), an etiological factor of leprosy and non-tuberculous mycobacteria (NTM) causing other mycobacterioses, has also been implicated in increased cancer risk [64,94,95,96]. Ratoosh et al. reported an association between cutaneous malignancy and leprosy, suggesting that the coexistence of M. leprae and cancer in the same lesion is likely secondary to the present high bacterial load [94]. A study by Lande et al. showed high rates of lung squamous cell carcinoma among people with previous or coexisting lung infection with the Mycobacterium avium (M. avium) complex (MAC) [64]. A case of Mycobacterium xenopi infection in a patient with squamous cell carcinoma was also reported [97]. The chronic persistence of M. avium subsp. paratuberculosis, the cause of chronic idiopathic inflammatory bowel disease (IIBD), in intestine was found to be correlated with the development of IIBD-associated and sporadic colorectal cancer [98]. Mycobacterium ulcerans infections were found to be correlated with scar carcinogenesis in the skin and other organs, while chronic Mycobacterium marinum infections acted as tumor promoters in liver tissues of Japanese medaka fish [73,95]. Although much scientific evidence suggests that chronic mycobacterial infections are closely related to tumorigenesis, their causal relationship remains controversial. There is still an unanswered question as to whether the chronic inflammatory environment created by the infection might lead to the development of cancer or whether lung cancer could allow the development of mycobacterial infection. Both scenarios are likely true, but determining which one happens more often may be a challenge.

*M.tb* can affect other organs as well as the lungs. Five cases of primary liver malignancy co-existing with isolated hepatic TB have been reported so far [99]. It is suggested that mycobacteria-induced reactive forms of oxygen species can damage cell DNA and lead to the development of cancer. Moreover, purified protein derivative (PPD) of *M.tb* can upregulate the expression of vascular endothelial growth factor in lymphocytes, which has significant angiogenic and mitogenic properties [99]. On the other hand, cancer treatment can also contribute to the development of TB [100]. Transarterial chemoembolization in hepatocellular carcinoma and cirrhosis may result in the development of symptomatic active TB. In patients who have reactivated TB, coexisting diseases may be the greatest factor, but cirrhosis is the main factor in the development of active disease [101]. The use of a PD-1 inhibitor in the treatment of neoplasms, including melanoma, also influences the development of TB. Unfortunately, this problem has not been resolved. In murine models, a decrease in the number of TB antigen-specific T cells was observed, which may be related to the effect of inhibitors on infection [102]. In addition, the life of mice was shortened and severe lung necrosis was observed [103]. However, anti-TB therapy also poses a risk of developing liver cancer if cirrhosis occurs [104]. This explains the necessity of diagnosing patients with TB before starting anticancer treatment [100].

## 5. Mycobacteria as Therapeutic Agents

Although bacteria are mostly considered pathogens, it was already suspected over 150 years ago that they might be useful in the treatment of cancer. This hypothesis was put forward independently by two German doctors, W. Bush and F. Fehleisen, when they noticed regression of neoplastic tumors in hospitalized patients accidentally infected with Streptococcus pyogenes [105]. The use of microbes in cancer therapy dates back to the 19th century when Dr. William Coley (1862–1936) developed a mixture of bacterial microbes and, for the first time in the history of modern medicine, successfully treated certain types of cancer, which led him to become a pioneer of immunotherapy [106]. Knowing about the spontaneous regression of sarcomas in patients with severe bacterial infection, he undertook the development of an experimental therapy to administer Streptococcus pyogenes to a patient with inoperable bone sarcoma. The results were extremely promising as the patient was diagnosed with complete tumor regression [107].

The first microorganism that was widely used in the fight against cancer was Mycobacterium bovis (M. bovis) BCG. Studies carried out in the late 1970s showed that intravesical administration of these bacteria reduces the risk of recurrence of non-muscle invasive bladder cancer. Infusions of the suspension of these mycobacteria are currently considered as a standard element of therapy in the oncological treatment of high–intermediate risk cancer, where the exact immune mechanism underlying this therapy is not entirely clear, and the pattern and timing of bacterial administration is the subject of ongoing testing [108]. Bacille Calmette–Guérin (BCG) is a commonly used vaccine for the prevention of TB. The name of the vaccine is a combination of the former name of the mycobacteria and the names of the French scientists who developed the vaccine—Albert Calmette and Camille Guérin. BCG is an attenuated strain of M. bovis (bovine mycobacterium) obtained at the Pasteur Institute in Lille, France. Calmette and Guérin systematically studied the passage of bacteria on a culture medium containing potato, glycerol, and bile. After 231 passages and 13 years of work, they obtained a strain that lost its pathogenic properties and could be used for vaccination. The vaccine was applied for the first time in 1920, and originally it was administrated orally. Analyses conducted in Belgium and France in the first years after the introduction of BCG showed that the vaccine was extremely effective in children [109,110,111].

Upon entry into the host organism, the pathogen provokes a response from the cells of the immune system. The presence of mycobacteria induces a response from both innate and acquired immune cells. Innate immunity is based on cells constantly circulating in the bloodstream, such as monocytes, natural killer (NK) cells, or neutrophils, as well as macrophages and dendritic cells. They recognize the antigens of the pathogen through specific receptors, which provoke a cascade of reactions, such as the release of cytokines or phagocytosis. On the other hand, acquired immunity is based on the process of pathogen recognition by lymphocytes, which among others consequently leads to the production of antibodies against this pathogen and the formation of memory cells storing a “picture” of its antigens. Vaccination is based on this process [112]. The BCG vaccine is also known for its non-specific properties [113,114,115,116]. Monocytes previously stimulated with BCG can respond more effectively when reinfected with unrelated microorganisms [117,118]. This effect is most associated with two immune mechanisms. The first depends on the epigenetic reprogramming of innate immune cells during a process termed trained immunity. The second is related to heterological responses from Th1 and Th17 lymphocytes and cross-protection [119,120,121]. Recent research calls into question the paradigm that innate immunity is completely devoid of adaptability. The non-specific beneficial effects of vaccines are increasingly being presented and cannot be explained solely by the acquired immunity mechanisms. This is also indicated by studies on plants and invertebrates, which are completely devoid of acquired immunity and show protective immune mechanisms after vaccine application or exposure to infections. These observations lead to the hypothesis that certain infections or vaccines can induce the reprogramming of congenital response mechanisms, leading to the development of non-specific protection against reinfection [112]. The BCG vaccine may play an important role in other cases as well. The results of many clinical trials show the beneficial effects of the use of the BCG vaccine as a first-line drug in the case of non-muscle invasive bladder cancer (NMIBC) to inhibit progression to muscle-invasive disease and to prevent recurrence of the disease. Unfortunately, data from many randomized clinical trials do not provide clear dose guidelines or frequency of vaccine administration [122]. In 1929, Pearl reported in the article “Cancer and tuberculosis” the potential anti-cancer effects of infection caused by tubercle bacilli. The data came from autopsies he conducted at Johns Hopkins Hospital, during which he noted that TB patients had significantly fewer malignancies compared to people who had no TB [123]. The first well-documented study confirming the anti-bladder cancer effect of BCG was conducted by Lamm in 1980 [124,125]. Lamm observed the development of the disease in 92 randomized patients and an additional 30 high-risk patients in their 5-year study [125]. Only 20% of patients given BCG as bladder infusion reported a relapse compared to 52% in the control group. However, during studies, cases of complications after BCG therapy, manifested by the formation of unwanted tuberculous granulomas in the liver, have been reported and described [126], and recent observations indicate that the use of immunotherapy with immune checkpoint inhibitors (ICIs) may drastically increase the risk of developing infectious diseases, including those caused by Mycobacterium avium complex bacilli [127]. Not only mycobacteria can participate in anti-cancer therapy. The use of Salmonella species in cancer immunotherapy is also a subject of interest. Like mycobacteria, these bacteria are intracellular pathogens. They penetrate the cells, multiplying and causing the death of infected host cells. Using mice as the animal model, it was shown that due to the retention of bacteria in the tumor and infection of the cancer cells, the growth of the tumor was stopped, and even its elimination was noticed. The first clinical trials were undertaken, which unfortunately led to disappointment for scientists. In the patient organism, such a distinct advantage of the neoplastic localization of microorganisms over the rest of the tissues was not observed. Therefore, it was necessary to modify the bacteria so that they prefer cancerous tissues in the human body. Scientists from the Faculty of Biochemistry, Biophysics, and Biotechnology at Jagiellonian University, Poland, modified the disposition of Salmonella, due to which it could be used for anti-cancer therapy. It expressed carcinoembryonic antigen (CEA)-specific single-chain antibody fragments to increase tumor-specific targeting and produced more SipB (Salmonella invasion protein), which increased the ability of bacteria to destroy infected cells [128].

How BCG immunotherapy affects the tumor is still not fully understood. Studies in animal models have shown that BCG interacts with the epithelium lining the bladder. Certain surface structures of the bacterial cell wall interact with fibronectin in the epithelium. Two possible scenarios of this interaction have been described. The first one indicates a physicochemical interaction that damages the glycosaminoglycan layer and provides BCG with easier access to the bladder wall and facilitates binding to fibronectin. The second scenario concerns specific receptor/ligand binding via fibronectin. It seems the presence of antigen 85 and fibronectin attachment protein (FAP) is required for BCG retention and targeting cells [129]. After the introduction of BCG into the bladder, the activation of epithelial cells and antigen-presenting cells that produce cytokines and chemokines is observed. As a result, granulocytes and mononuclear cells begin to flow into the bladder. After the intravesical administration of BCG, the appearance of macrophages, dendritic cells, lymphocytes, and neutrophils is observed in the bladder wall [129]. In vitro studies with the use of human NIMBC cancer cell lines showed that BCG increases the production of IL-6 and IL-8, GM-CSF (granulocyte-macrophage colony-stimulating factor), and TNF. Furthermore, data obtained from studies on human subjects showed that after the introduction of BCG, neutrophils, macrophages, monocytes, T and B lymphocytes, and NK cells are present in the bladder, accompanied by increased levels of IL-1β, IL-8, IL-15, IL-18, CXCL10 (CXC motif chemokine ligand 10), CC motif chemokine ligand (CCL)2, CCL3, and GM-CSF. Neutrophils can directly affect tumor cells through their phagocytic activity and the ability to produce reactive oxygen species, secretion of lytic enzymes, and factors inducing apoptosis, e.g., TRAIL (TNF-related apoptosis-inducing ligand). In the case of successful immunotherapy, intensive secretion of IL-2, IL-12, IFN-γ, TNF-α, and TNF-β was observed, whereas in the case of failure the production of IL-4, IL-5, IL-6, and IL-10 was noticed [129].

Although the immune response initiated by BCG reduces the risk of recurrence and progression of bladder cancer, this therapy is not always effective. The reason for this situation has not yet been found. It cannot be ruled out that the diversity of BCG strains may play a role here. After BCG was developed and shipped around the world, it was cultured for decades under slightly different conditions, which resulted in the generation of several different substrains. A few daughter strains derived directly from the parental BCG have been identified, and they are characterized mainly by a deletion of the region of differentiation (RD) 1. The daughter strain group includes BCG Russia, Moreau, Japan, Sweden, and Birkhaug. Moreover, some “late” BCG substrains have been established (Prague, Glaxo, Danish, Tice, Frappier, Connaught, Phipps, Pasteur), which are generally distinguished by the elimination of RD2 [130]. The substrains differ especially in the composition and the number of certain components of their cell wall, which applies especially to mycolic acids, phthiocerol dimycocerosates (PDIM), and phenolic glycolipids (PGLs)—lipids that play a particular role in microbe–host interactions [130]. Although the majority of studies indicate the lack of advantage of any of the BCG substrains over the others in terms of effectiveness in the therapy of nonmuscle-invasive bladder cancer (NMIBC) [131,132,133], some reports indicate such differences. Comparing the effectiveness of the two commonly used BCG strains in the treatment of NMIBC Connaught and Tice, a significant difference in the length of the relapse-free period was observed in favor of the former, indicating a difference in the immunogenicity of the two strains [134]. Experiments on mice C57BL/6 showed that the Connaught strain induced greater priming of BCG-specific CD8+ T cells and caused stronger recruitment of CD45+ leukocytes, NK cells, NKT cells, CD3+ T cells, CD4+ T cells, and CD8+ T cells to the bladder than the Tice strain. It cannot be excluded that single nucleotide polymorphisms (SNPs) of BCG strains may be responsible, at least partially, for the differential host response. Sequencing of the strains revealed genetic loss of region RD15 in the Connaught strain and 12 SNPs common to Connaught and Tice strains. Two SNPs and one small insertion and deletion (INDEL) were present exclusively in the BCG Tice strain (not in the other BCG strains or *M.tb*). One of the SNPs and the INDEL influence the open reading frames of BCG_3474c, BCG_2151c proteins of unknown function, while the second SNP is in the gene encoding the copper–zinc superoxide dismutase C (sodC) and is involved in a nonsynonymous change from the highly conserved CAC (His) codon to a CAG (Gln) codon [134].

## 6. Conclusions

**Figure 2:**
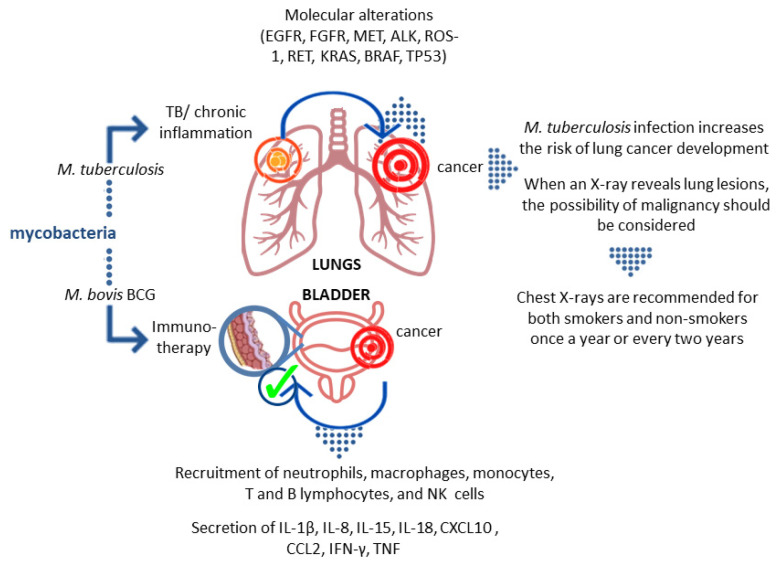
The dual nature of the relationship between mycobacteria and cancer. The risk of tumor development in chronic *M.tb* infections is believed to be the result of multiple host effector mechanisms at various stages of oncogenesis. On the other hand, the influence of *M. bovis* BCG on the immune system prevents the development of neoplastic processes and/or increases the effectiveness of cancer therapy.

Some indications point out the link between lung cancer development and *M.tb* infection (Figure 2). The role of *M.tb*-induced inflammatory events in the development of lung cancer is still being intensively studied [59,60,76,77,78,79,80]. Interestingly, another member of the genus *Mycobacterium*—*M. bovis* BCG—shows anti-tumor properties [123,124,125]. Mycobacterial suspension infusions are a standard therapy element in the oncological treatment of intermediate-risk bladder cancer [108]. The influence of mycobacteria on the immune system still hides many unclear issues, and recognizing and explaining them may prevent the development of neoplastic processes and/or make anticancer therapy more successful. It is widely known that infected individuals do not experience lung pain due to the lack of sensory innervation, and hence many lung diseases remain asymptomatic for a long time. This applies particularly to such diseases as TB and lung cancer. For this reason, and because of the worse epidemiological situation related to TB, until the 1990s, annual chest examinations were obligatory in Poland, either in the form of radiographic photography (photofluorography) or a standard X-ray image of the chest; this obligation was later abolished. From the perspective of over two decades, the consequence of this decision, in the opinion of many medical practitioners, is a delay in TB and lung cancer diagnosis. Therefore, it is increasingly postulated that non-smokers should have a chest X-ray at least once every two years, and smokers should have a prophylactic chest checkup at least once a year, which would be in the best interest of the patient.

## Data Availability

The data presented in this study are openly available under reference numbers.
